# Repeated Fed-Batch Culture Strategy for the Synthesis of Polyhydroxybutyrate (PHB) Biopolymers from Sugar Cane Juice Using *Azotobacter vinelandii*

**DOI:** 10.3390/polym16223156

**Published:** 2024-11-13

**Authors:** Praepilas Dujjanutat, Pakjirat Singhaboot, Pakawadee Kaewkannetra

**Affiliations:** 1 Postdoctural Training of Department of Biotechnology, Faculty of Technology, Khon Kaen University, Khon Kaen 40002, Thailand; praedujja@gamil.com; 2 Faculty of Agricultural Product Innovation and Technology, Srinakharinwirot University, Nakhon Nayok 26120, Thailand; pakjirat@g.swu.ac.th; 3 Department of Biotechnology, Faculty of Technology, Khon Kaen University, Khon Kaen 40002, Thailand

**Keywords:** polyhydroxyalkanoates (PHAs), polyhydroxybutyrate (PHB), *Azotobacter vinelandii*, sugar cane juice (SCJ), repeated fed-batch fermentation

## Abstract

In this research work, a main biopolymer group of polyhydroxyalkanoates (PHAs) in the form of polyhydroxybutyrate (PHB) was synthesised by a pure bacterial strain of *Azotobacter vinelandii* via repeated fed-batch fermentation. An agricultural crop, sugar cane, was used as the sole carbon source. Firstly, batch fermentation was investigated considering variations in incubation times (24 h, 48 h, and 96 h). The highest dry cell weight (DCW) and PHAs of 5.15 ± 0.04 g/L and 4.00 ± 0.04 g/L were obtained after 48 h of incubation time. The optimum time obtained was further varied to investigate the effect of the sugar concentrations in the medium. It was found that bacteria could grow very well and produced the highest DCW and PHAs (11.17 ± 0.15 g/L and 8.77 ± 0.06 g/L) when the culture medium with a 100 g/L sugar concentration was added. Later, repeated fed-batch fermentation was carried out to improve productivity. The results obtained revealed that PHA production was increased in the next cycle of the process. Furthermore, the final productivity (0.104 g/L·h) was increased 1.65-fold compared to the first cycle (0.063 g/L·h). Moreover, the culture strategy showed remarkable results, with reductions in both fermentation time and preparation cost.

## 1. Introduction

Polyhydroxyalkanoates (PHAs), one of the main kinds of organic biopolymer, are aliphatic polyesters produced completely naturally by numerous microorganisms, including yeast, fungi, and, predominantly, bacteria, via the fermentation of sugars and lipids as raw materials [1,2,3]. Naturally, in this case, various microorganisms produce PHAs as carbon and energy sources under imbalanced conditions with excess carbon sources and limited amounts of some nutrients, such as nitrogen, phosphorous, potassium, magnesium, or sulphur, and also under environmental stress conditions such as ones involving limited oxygen [4,5,6]. Currently, PHAs are very interesting to most researchers and industrial sectors because of their potential conversion into biodegradable thermoplastics and their properties, which are similar to those of petroleum synthetic plastics such as polyethylene (PE) and polypropylene (PP) [7]. Moreover, they can be completely degraded in the environment [8,9]. Therefore, PHAs are used to produce biodegradable products for a wide range of applications in areas such as packaging, medical equipment, and disposable personal hygiene [10]. 

Typically, different sugars such as glucose, fructose, and even sucrose are suitable for use as carbon sources in PHA production. Previous studies have attempted to produce PHAs using several sugar-based crops, which are used as raw materials, for example, sugar cane in the forms of juice and bagasse [11,12,13], sweet sorghum [14,15], maple sap [16], sugar beet molasses [17,18], and, recently, pineapple cannery waste [2]. According to sugar cane experts, it is one of the important economic crops, alongside rice, natural rubber, and others. It is widely grown throughout Thailand, especially in the northeastern region. Therefore, in this work, sugar cane juice (SCJ) was chosen as a carbon source for the production of PHAs. Some physicochemical properties of SCJ were characterised and we showed that the sugar contained a very high sucrose concentration. Furthermore, the juices from other sugars such as glucose and fructose were also found in small amounts and less frequently than sucrose. 

Among monomers of PHAs, polyhydroxybutyrate (PHB), which has a non-complex structure and contains only one monomer of 3-hydroxybutrarate (3-HB), is usually found, shows the best characteristics, and is also widely produced by many bacterial strains, including *Alcaligenes eutrophus*, *Bacillus aryabhattai* PKV01, and *Alcaligenes latus*, which are now known as *Azohydromonas lata*, *Azotobacter vinelandii*, and *Bacillus licheniformis* NS032 [11,12,13,14,17,18,19]. However, a bacterial strain of *A. vinelandii* was chosen to study PHA production in this research because it mainly utilises sucrose as a carbon source. Furthermore, our group has used the strain to ferment SCJ under fed-batch fermentation conditions. Naturally, *A. vinelandii* is a Gram-negative, obligate aerobe able to fix nitrogen and to adapt its metabolism to diverse environmental conditions [19,20]. It is known for producing alginate and siderophores. Moreover, it can produce PHAs during growth under imbalanced conditions and it accumulates P(3HB), forming up to 75% of its dry cell mass during exponential growth [20]. Previous works reported the production of PHAs by *A. vinelandii* using sugar beet molasses as a raw material. The results showed that the highest PHAs concentration and productivity were 22 g/L and 0.55 g/L·h, respectively [17,18]. 

Most studies on PHA production by *A. vinelandii* have been performed based on batch fermentation because of its easy process, the lack of a need to refill nutrients after starting the process, and the product yield at the end of the process. However, the batch process often encounters the substrate inhibition problem when a substrate with a high concentration is used. Therefore, fed-batch fermentation, in which a base medium supports the initial cell culture, is usually used as an alternative process to solve the substrate inhibition problem because substrates are supplied to the system during cultivation and the product remains in the system until the end of the run. In repeated fed-batch fermentation, its typical long duration and culture stability are important factors. The process is similar to fed-batch fermentation, but only at the end of each cycle. Fermentation broth is withdrawn and remains just a partial broth for use as a seed culture in the next cycle, and the addition of fresh medium into the fermentor is performed. As a result, it reduces the energy used and the time needed for the preparation of the equipment. 

Repeated fed-batch fermentation has been successfully used to improve the performances of other processes such as batch, fed-batch, and repeated batch modes. For example, previous works were reported that the effective production of DHA-rich microbial lipids via different fermentation processes including batch, fed-batch, and repeated fed-batch processes by *Schizochytrium* sp. The fed-batch process was a more efficient method of cultivation than both the batch and repeated fed-batch processes [21,22]. Further more, it also showed more advantages, such as reductions in time, cost for seed culture, and inoculation in each fermentation cycle. For example, the enhancing efficiency of acetic acid production by using repeated fed-batch fermentation [23]. In other studies, researchers used repeated fed-batch processes to improve the efficiency of many fermentation products such as ethanol [24], xylitol [25], lipase [26], dihydroxyacetone [27], and rhamnolipid [28]. However, for the production of PHAs using repeated fed-batch fermentation, there is no study reported. Only a repeated batch fermentation of PHB by *R. eutropha* was found. The total PHB productivity obtained at the end of 67 h was 0.42 g/L·h. As compared to the batch process, two cycles of feeding led to a three-fold increase in PHB productivity [29]. 

Therefore, PHA production by repeated fed-batch fermentation will be the focus for this study. Some effects of fed-batch fermentation on cell growth and PHA production by *A. vinelandii* when SCJ is used as a carbon source were examined. An investigation of their stability in a repeated fed-batch fermentation process and an evaluation of an effective process in terms of production yield and productivity were considered. In addition, a physicochemical property of PHAs was also evaluated.

## 2. Materials and Methods

### 2.1. Bacterial Strain and Initial Inoculum Preparation

A pure bacterial strain of *A. vinelandii* was kindly provided by the Thailand Institute of Scientific and Technological Research (TISTR), Bangkok, Thailand. The culture was then sub-cultured and maintained at 4 °C and transferred monthly on Burk’s agar containing 3 g/L beef extract, 5 g/L peptone, and 15 g/L agar.

To prepare inoculums, a loopful of *A. vinelandii* cells was transferred to an Erlenmeyer flask (250 mL) and grown in Burk’s medium (Sigma-Aldrich, Hamburg, Germany), a free nitrogen medium used for the isolation and cultivation of nitrogen-fixing bacteria from soil, containing (g/L) 0.64 K_2_HPO_4_; 0.20 KH_2_PO_4_; 0.2 MgSO_4_·7H_2_O; 0.2 NaCl; 0.05 CaSO_4_·2H_2_O; 20 mannitol; 0.001 Na_2_MoO_4_, and 0.003 FeSO_4_. Then, the pH of the culture medium was adjusted to be 7.0, and the medium was incubated on a shaker at 30 °C, 200 rpm, for 48 h. After that, 10% (*v*/*v*) of the culture was transferred into fresh Burk’s medium for 24–30 h. The culture was then ready to use as initial inoculums for the next experiments.

### 2.2. Preparation of Sugar Cane Juice (SCJ)

Only sugar cane juice was used as a carbon source for producing PHAs. Parts of sugar cane stems were collected and harvested from a sugar cane plantation in the northeastern areas of Khon Kaen province, Thailand. Then, they were squeezed for the juice by roller mills. The SCJ was filtered by cotton sheet and then kept at −20 °C to prevent microbial contamination. It was left at room temperature and then was sterilised at a low temperature of 62.8 °C for 30 min prior to use. 

### 2.3. Fed-Batch Fermentation

#### 2.3.1. Variations of Fermentation Times

Burk’s medium with the SCJ as a carbon source (initial sugar of 20 g/L) and ammonium acetate (1.2 g/L) as a nitrogen source was prepared for the evaluation of the growth ability of and PHAs produced by *A. vinelandii*. Experiments were carried out in a 500 mL Erlenmeyer flask containing 200 mL of culture medium. These flasks were inoculated with 10% (*v*/*v*) initial inoculums and incubated at 30 °C on a shaker (200 rpm). Then, 100 mL fresh medium with the SCJ (sugar concentration of 40 g/L) was added into flasks after incubation times of 24 h, 48 h, and 96 h. Samples were withdrawn during fermentation for analysis of the dry cell weight (DCW), PHAs, and residual total sugar concentrations. The optimal fermentation time (the condition with the maximum DCW and PHAs) was used for further experiments.

#### 2.3.2. Variations of Sugar Concentrations in Culture Medium

The total sugar concentrations of the productive medium were varied between 60, 80, and 100 g/L. The effect of the sugar concentrations of the feed medium was considered in a 5 L fermentor with an initial working volume of 1 L. Initial inoculums (10% *v*/*v*) were inoculated into the production medium with the SCJ as a carbon source and ammonium acetate as a nitrogen source. Temperature and pH were constantly maintained at 30 °C and 7 during fermentation. The air supply into the system was fed continuously at a rate of 2 vvm via air pump and speed of agitation rate of 250 rpm. After achieving the optimum time obtained from the previous experiment, the culture medium was added into the fermentation system to start the fed-batch mode. The final working volume was made up to 2.5 L. Some parameters, the DCW, PHAs, and residual total sugar concentrations, were analysed during fermentation. 

### 2.4. Repeated Fed-Batch Fermentation

The best condition of the previous experiments was used to investigate a repeated fed-batch fermentation. The process performed similarly to a fed-batch fermentation. The differences from the fed-batch culture were that the fermentation broth was withdrawn at the end of each fermentation cycle and about 10% broth remained for use as a seed culture in the next cycle. In addition, fresh production medium was also added to obtain a final concentration of total sugar in the broth of 20 g/L. 

### 2.5. Recovery of PHA Product and Its Characteristic

PHAs accumulated as an intracellular product, and, therefore, the presence of PHAs was confirmed by the Sudan black B staining method. The modified gravimetrical method described by Chala et al. (2019) [30] was used to determine the extracted PHAs. The sequence steps were as follows: the wet cell mass or cell pellet obtained from centrifugation was treated with 3% (*v*/*v*) sodium hypochlorite (commercial grade bleach), incubated at 37 °C, and stirred for 1 h. Thereafter, chloroform was added into the mixed solution, and it was then stirred at 50 °C ± 5 °C for 3 h. An upper phase of chloroform was separated and evapourated. Then, the white powder/sheet was left at room temperature until it reached a constant weight. The extracted PHAs were measured by weighing. The physicochemical property of the PHAs film produced was analysed using Fourier Transform Infrared Spectroscopy (FTIR). Spectrograms were scanned between 4000 and 6000 cm^−1^. The FITR result was compared to the polymer standard reagent (analytical grade) of polyhydroxybutyrate (PHB) (Sigma Aldrich, St. Louis, MO, USA). 

### 2.6. Analytical Techniques

To ascertain the characteristics of the SCJ, all compositions were analysed using standard methods. For example, the sugar contents, 3 elements (calcium, magnesium, and zinc), phosphorus, nitrogen, and total soluble solids (TTS) were measured using a hand refractometer (Hanna Instrument, Bangkok, Thailand), an Atomic Absorption Spectro-photometer (AAS) (BUCK Scientific, Ansonia, CT, USA), the Vannadomolydate method, the Kjeldahl method (Gerhardt, Brackley, UK), and High-Performance Liquid Chroma-tography (HPLC) (Shimadzu, Tokyo, Japan), respectively.

During fermentation, for biomass analysis, cell growth was monitored by the measurement of the optical density at 600 nm (*A*_600_) using a spectrophotometer (PG Instruments Limited, T60, Lutterworth, UK). The culture broth was centrifuged at 10,000× *g* for 10 min. The supernatant was separated, and only the pellets were washed twice with deionised water, and then were dried in a hot air oven (80 °C) until they reached a constant weight. Then, dried pellets were used for DCW analysis, while the supernatant was analysed for residual total sugar contents using HPLC with a refractive index detector (RID), a Vertical GES-NH_2_ HPLC column (4.6 × 250 mm, 5 μm), and 75% acetonitrile in deionised water as the mobile phase. The flow rate at 1 mL/min and the injection volume were controlled at 20 μL. In addition, a rapid Sudan black B staining method was used to monitor an intracellular product of the PHAs. After fermentation, the PHA product was recovered using a series of downstream methods including precipitation, disrubtion, and extraction [12]. The physical property of the PHA film sheet was analysed using the Fourier Transform Infrared Spectroscopy (FTIR) technique (Bruker TENSOR 27, Spectra Lab, Toronto, ON, Canada).

## 3. Results and Discussion

### 3.1. Characteristics of Sugar Cane Juice (SCJ)

Some physical and chemical properties of the SCJ were characterised, as shown in Table 1. Total sugar was found to have a very high concentration (216.10 g/L) and consisted of three different sugars (sucrose, glucose, and fructose). Sucrose was mainly found in quite a high concentration (approximately 202 g/L). The total sugar found in the current study was higher than that found in our previous work (201.64 g/L); although the sugar cane stems were collected in the same location, they were collected at different times [11]. For elements, the main element of potassium (K), was found in a high concentration at about 5523 mg/L, while other elements, such as sodium (Na), calcium (Ca), magnesium (Mg), and zinc (Zn), were also found in small concentrations. The degree Brix (°Brx) represents the sugar content of an aqueous solution. It should be noted that one degree Brix means 1 g sucrose in 100 g of water and expresses that the concentration of the solution as a percentage of 1 g sucrose in 99 g solution (liquid). However, the SCJ contains a large amount of sugar and other components. Thus, the SCJ found is different depending on several factors such as season, location, plantation area, etc. We have analysed the SCJ that was obtained from the same location (Khon Kaen province) but different plantation areas. It was found that the SCJ properties were quite similar [11,12,13]. 

### 3.2. Variations of Incubation Time in Fed-Batch Fermentation

**Figure 1:**
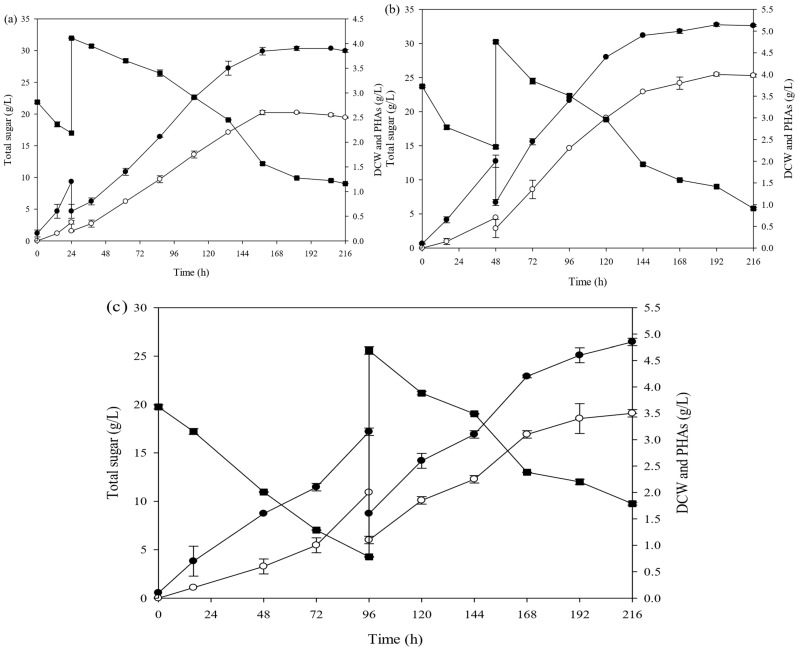
Monitoring of DCW ( ● ), total sugar ( ■ ), and PHA production ( ○ ) by *A. vinelandii* as a function of times under fed-batch fermentation after addition of the production medium at 24 h (**a**), 48 h (**b**), and 96 h (**c**).

The effect of incubation time in batch mode was studied at flask scale. In our previous work, we studied the production of PHAs by batch fermentation [11]. The growth profile of *A. vinelandii* via batch fermentation was used for this study. The batch profile showed that *A. vinelandii* could grow into a lag phase at about 24 h. After 48 h, it reached the mid-log phase, and at about 96 h, it entered into the early stationary phase. Therefore, incubation times (24 h, 48 h, and 96 h) were varied during operation in batch mode before conversion to fed-batch fermentation. When the fed-batch fermentation was started, the production medium with 40 g/L sugar concentration was added into the system. In Figure 1, the total sugar, dry cell weight (DCW), and PHA production during fed-batch fermentation after addition of the production medium at (a) 24 h, (b) 48 h, and (c) 96 h are shown.

**Figure 2:**
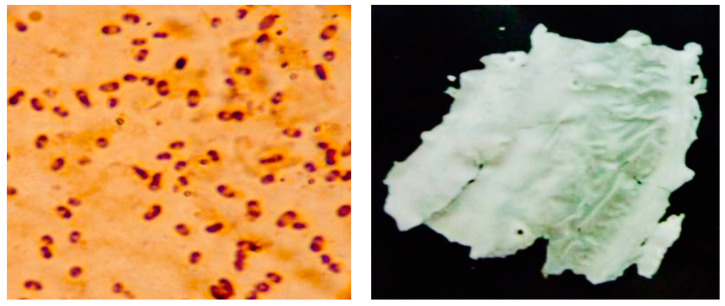
(**left**) PHAs accumulated in cells (100×) obtained by Sudan black B staining method at 48 h; (**right**) PHA sheet recovery produced by *A*. *vinelandii* after fermentation.

An intracellular PHA product synthesised by *A*. *Vinelandii* was also preliminarily confirmed by the Sudan black B staining method, as shown in Figure 2 (left). Meanwhile, after fermentation, the white PHA film product was recovered using downstream processes such as precipitation, disruption, and extraction following the procedures explained by Suwannasing et al. in 2015 [12], as shown in Figure 2 (right).

**Figure 3:**
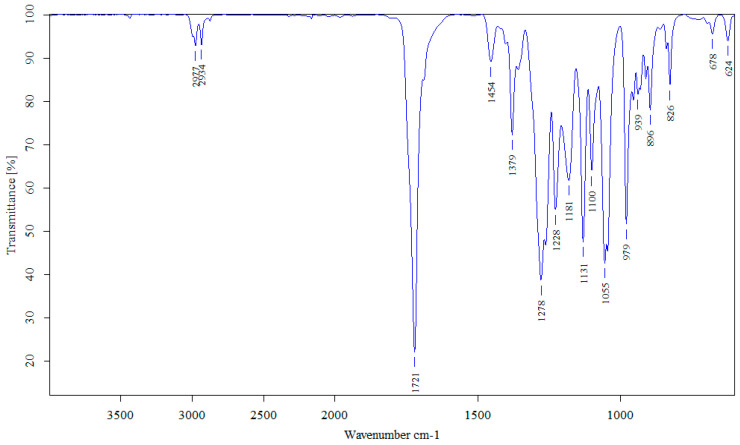

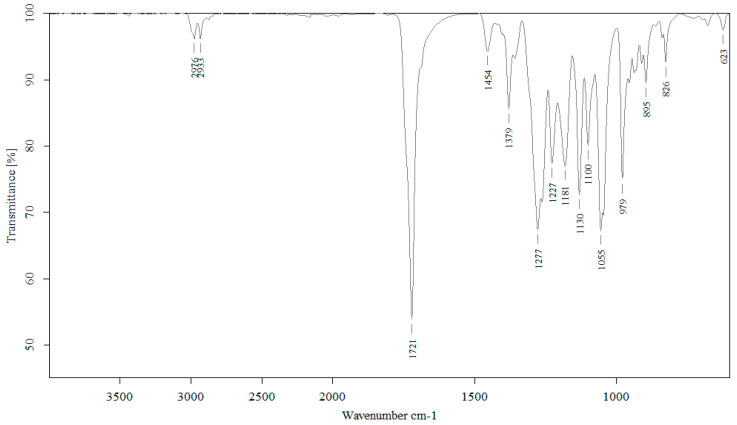
(**upper**) FTIR spectrum obtained from PHB (analytical grade); (**lower**) PHAs produced by *A*. *vinelandii*.

The molecule was identified on the wavelength measured using the FTIR spectrophotometer, which scanned between 600 cm^−1^ and 4000 cm^−1^. The peaks at 1721 cm^−1^ and 1277 cm^−1^ corresponded to the C=O and –CH group (see Figure 3). The FTIR spectrum of PHAs obtained from SCJ fermentation was in full agreement with those obtained from a standard sample of polyhydroxybutyrate (PHB). The results indicated that PHAs from SCJ could be characterised as a PHB. The FTIR spectrogram showed similar results to those reported in previous studies [12,13,31,32].

### 3.3. Variations of Sugar Concentrations in Culture Medium Under Fed-Batch Fermentation 

**Figure 4:**
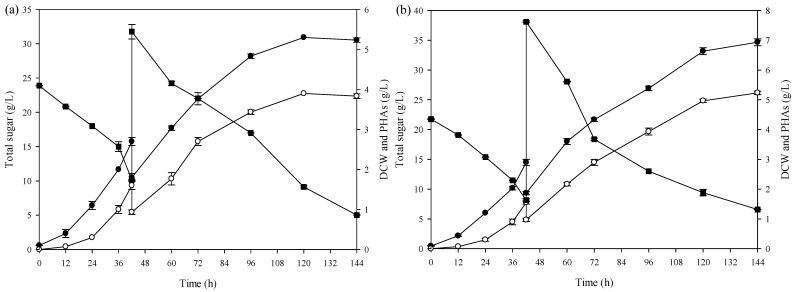

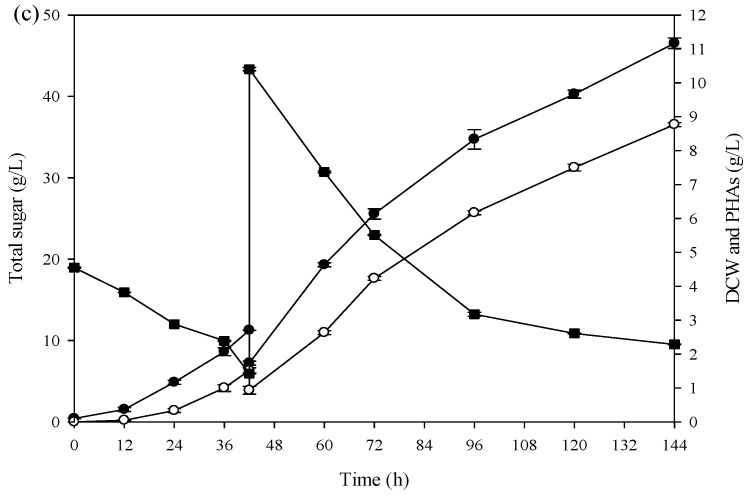
Fermentation kinetics growth profile and PHAs production by *A. vinelandii* in different sugar concentrations in culture medium during fed-batch fermentation: (**a**) 60 g/L, (**b**) 80 g/L, and (**c**) 100 g/L, (DCW ( ● ), total sugar ( ■ ), PHAs production ( ○ ).

The optimum incubation time in batch mode was used to study the effect of sugar concentration in feed medium on PHA production for 48 h in a 5 L bioreactor. The batch profile *of A. vinelandii* in the 5 L bioreactor revealed that the bacteria reached a mid-log phase at about 42 h under the conditions of 30 °C, 250 rpm, pH 7, and aeration rate 2 vvm. Then, culture media containing sugar concentrations of 60, 80, and 100 g/L were separately added into the bioreactors. It was found that the DCW and PHAs were increased as sugar concentrations increased [11]. The highest DCW and PHA concentrations of 11.17 ± 0.15 g/L and 8.77 ± 0.06 g/L were obtained from the concentration of 100 g/L. The growth profile is shown in Figure 4.

### 3.4. Repeated Fed-Batch Fermentation

The production of PHAs by the repeated fed-batch fermentation process was similar to that of the fed-batch fermentation process. Only at the end of the repeated fed-batch mode, the partial culture broth was used as a starter for the next cycle. The optimum conditions from the previous experiments were applied for the repeated fed-batch fermentation. In brief, for cycle 1 of the repeated fed-batch fermentation, 1000 mL of Burk’s medium with the SCJ as a carbon source (total sugar of 100 g/L) and ammonium acetate (1.2 g/L) as a nitrogen source was added into the bioreactor after 42 h of cultivation, and the final working volume was 2.5 L. After 96 h of fermentation, the DCW and PHAs of 8.37 ± 0.06 g/L and 6.07 ± 0.12 g/L were obtained at the end of cycle 1. After quitting the process, a 10% volume of culture broth remained for seed culture in the following cycle. The biomass density of the starter in cycle 2 was greater than in the first cycle. The growth profile of *A. vinelandii* was faster than that found in cycle 1. Therefore, the medium was added after 36 h, and the process was terminated when it reached 84 h of cultivation time. The DCW and PHAs were obtained at 9.17 ± 0.06 g/L and 6.70 ± 0.70 g/L.

**Figure 5:**
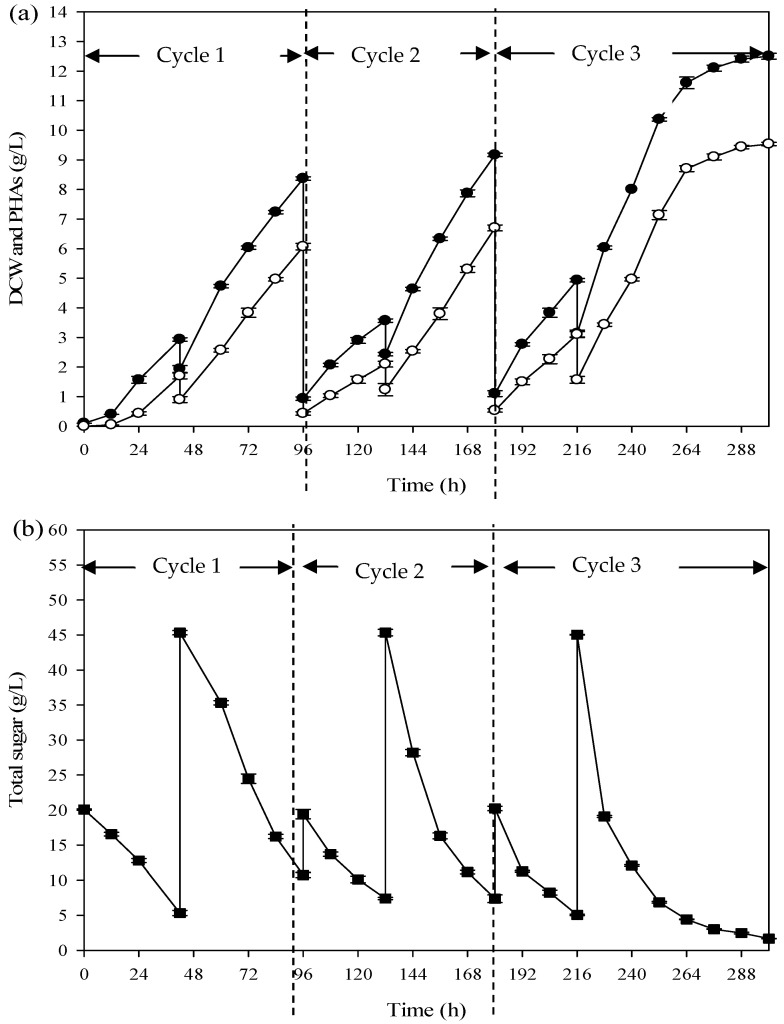
Kinetics of PHA production under repeated fed-batch fermentation of the SCJ by *A. vinelandii*: (**a**) DCW ( ● ) and PHA concentration ( ○ ); (**b**) total sugar ( ■ ).

Figure 5 shows the growth and PHA production profile of *A. vinelandii* during repeated fed-batch fermentation. As shown in (a), the method of experimentation in the final cycle was similar to cycle 2. In the last cycle, the DCW and PHAs reached 11.60 ± 0.20 g/L and 8.70 ± 0.10 g/L within 84 h incubation time. Meanwhile, (b) shows the sugar utilisation profile as a function of time for three cycles. For the first time, Burk’s medium with 20 g/L total sugar was used in batch mode. Then, medium with a 100 g/L concentration was loaded into the system. At the beginning of the next cycle, the fresh medium was added to the bioreactor and the final concentration was 20 g/L. After 36 h, the medium with a concentration of 100 g/L was loaded into the bioreactor, the same as in the first cycle. According to the profile, it can be stated that *A. vinelandii* shows a high efficiency in using the sugar in SCJ as a carbon source for growth and PHA production.

Table 2 shows some parameters for the three cycles of repeated fed-batch fermentation. It was clearly found that the fermentation time of the process was reduced in the next cycle. Moreover, the productivity in the final cycle (0.104 g/L·h) was increased by 1.65 folds when compared to the first cycle (0.063 g/L·h). In addition, the duration of time in each batch mode during operation of a repeated fed-batch fermentation was shorter than that found in the batch fermentation. In addition, compared to previous work [11], when fed-batch fermentation of SCJ by *A. vinelandii* was performed, both of the PHA contents and productivity obtained at 62.79%–70.83% for all cycles that were less than those of the repeated fed batch fermentation (72.51%–75.0%). 

The results obtained were similar and in agreement with previous studies. For example, Yang and Tsao (1995), who studied acetone–butanol fermentation using repeated fed-batch operation, found that the integrated process reached 47.2 g/L equivalent solvent concentration and 1.69 g/L·h fermenter productivity [33]. Compared to a controlled traditional batch acetone–butanol fermentation, the equivalent solvent concentration and the fermenter productivity were also increased. Novak et al. (1997) reported that lovastatin formation by a pure fugal strain of *Aspergillus terreus* increased, when the end in each cycle of repeated fed batch fermentation was increased. The yield of lovastatin was increased by 37%, though this took over twice as long as in the batch fermentation [34]. Kumar et al. (2000) also studied the repeated fed-batch process for improving lovastatin production. They found that this process could increase lovastatin yield, and the overall volumetric productivity was increased by 73% over the batch process [35].

## 4. Conclusions

Remarkably, the experimental results demonstrated that the SCJ could be used as a sole carbon source for bacterial growth and PHA production in the form of polyhydroxybutyrate (PHB). Naturally, the SCJ contained a high sugar content. Therefore, it could be diluted to prepare a raw material and could be reduced for a raw material cost. The production of PHAs by a repeated fed-batch process can be an alternative to the fermentation process due to an enhanced fermentation performance in terms of yield and productivity compared to batch, fed-batch and repeated batch processes. Moreover, the process overcame drawbacks typically associated with a batch operation such as fermentation time reduction, a long lag period, and limitation of the maximum initial substrate concentration due to substrate inhibition. In addition, the process of repeated fed-batch fermentation for other sugar-based agricultural raw materials, other bacterial strains, and even high-gravity/very-high-gravity (VHG) fermentations should be studied in further works.

## Figures and Tables

**Table 1:** Compositions of sugar cane juice.

Component		Concentration
**Sugar**	Sucrose	202 g/L
	Glucose	8.7 g/L
	Fructose	5.4 g/L
	Total sugar	216.10 g/L
**Elements**	Potassium (K)	5223 mg/L
	Sodium (Na)	8.1 mg/L
	Calcium (Ca)	21.43 mg/L
	Magnesium (Mg)	142 mg/L
	Zinc (Zn)	0.3 mg/L
Nitrogen % (*w*/*v*)		1.40
pH		5.7
Total soluble solids (°Brx)		22

**Table 2:** Comparison of some parameters monitored during repeated fed-batch fermentation (using SCJ as a carbon source).

Cycle	Time	DCW	PHAs	PHA Content	Productivity
	(h)	(g/L)	(g/L)	(%)	(g/L·h)
1	96	8.37 ± 0.06	6.07 ± 0.12	72.51	0.063
2	84	9.17 ± 0.06	6.70 ± 0.10	73.09	0.080
3	84	11.60 ± 0.20	8.70 ± 0.10	75.00	0.104

PHA content = PHAs produced per DCW × 100. PHA Productivity = PHA concentration produced per time.

## Data Availability

The original contributions presented in the study are included in the article, further inquiries can be directed to the corresponding author.

## References

[1] Surendran A., Lakshmanan M., Chee J.Y., Sulaiman A.M., Thuo D.V., Sudesh K. (2020). Can Polyhydroxyalkanoates be produced efficiently from waste plant and animal oils?. Front. Bioeng. Biotechnol..

[2] Suwannasing W., Tanamool V., Singhaboot P., Kaewkannetra P. (2023). Valorisation of Pineapple Cannery Waste as a Cost Effective Carbon Source for Poly 3-hydroxyabutyrate (P3HB) Production. Polymers.

[3] Poirier Y., Nawrath C., Somerville C. (1995). Production of polyhydroxyalkanoates, a family of biodegradable plastics and elastomers, in bacteria and plant. Biotechnology.

[4] Steinbuchel A., Byrom D. (1991). Polyhydroxyalkanoic acids. Biomaterials: Novel Materials from Biological Sources.

[5] Byrom D., Mobley D.P. (1994). Polyhydroxyalkanoates. Plastic from Microbes: Microbial Synthesis of Polymers and Polymer Precursors.

[6] Park S.J., Kim T.W., Kim M.K., Lee S.Y., Lim S.-C. (2012). Advanced bacterial polyhydroxyalkanoates: Towards a versatile and sustainable platform for unnatural tailor-made polyesters. Biotechnol. Adv..

[7] Barham P.J., Dawes E.A. (1990). Physical properties of poly (hydroxybutyrate) and poly (hydroxybutyrate-co-hydroxyvalerate). Novel Biodegradable Microbial Polymers.

[8] Luzier W.D. (1992). Materials derived from biomass/biodegradable. Proc. Natl. Acad. Sci. USA.

[9] Lee S.Y. (1996). Bacterial polyhydroxyalkanoates. Biotechnol. Bioeng..

[10] Ojumu T.V., Yu J., Solomon B.O. (2004). Production of Polyhydroxyalkanoates, a bacterial biodegradable polymer. Afr. J. Biotechnol..

[11] Singhaboot P., Kaewkannetra P. (2014). Effectiveness enhancement of sugar cane juice fermentation for polyhydroxyalkanoates (PHAs) production. Asia-Pac. J. Sci. Technol..

[12] Suwannasing W., Imai T., Kaewkannetra P. (2015). Cost-effective defined medium for the production of polyhydroxyalkanoates using agricultural raw materials. Bioresour Technol..

[13] Singhaboot P., Kaewkannetra P. (2015). A higher in value biopolymer product of polyhydroxyalkanoates (PHAs) synthesized by *Alcaligenes latus* in batch/repeated batch fermentation processes of sugar cane juice. Ann. Microbiol..

[14] Tanamool V., Imai T., Danvirutai P., Kaewkannetra P. (2013). An Alternative Approach to the Fermentation of Sweet Sorghum Juice into Biopolymer of Poly-*β*-hydroxyalkanoates (PHAs) by Newly Isolated, *Bacillus aryabhattai* PKV01. Biotechnol. Bioprocess Eng..

[15] Tanamool V., Imai T., Danvirutai P., Kaewkannetra P. (2013). Biopolymer generation from sweet sorghum juice: Screening, isolation, identification, and fermentative polyhydroxyalkanoate production by *Bacillus aryabhattai*. Turk. J. Biol..

[16] Yezza A., Halasz A., Levadoux W., Hawari J. (2007). Production of poly-β-hydroxybutyrate (PHB) by *Alcaligenes latus* from maple sap. Appl. Microbiol. Biotechnol..

[17] Page W.J. (1992). Production of polyhydroxyalkanoates by *Azotobacter vinelandii* UWD in beet molasses culture. FEMS Microbiol. Rev..

[18] Gojgic-Cvijovic G., Jakovljevic D., Loncarevic B., Todorovic N., Pergal M., Ciric J., Loos K., Beskoski V., Vrvic M. (2018). Production of levan by *Bacillus licheniformis* NS032 in sugar beet molasses-based medium. Int. J. Biol. Macromol..

[19] Diaz-Barrera A., Soto E. (2010). Biotechnological uses of *Azotobacter vinelandii*: Current state, limits and prospects. Afr. J. Biotechnol..

[20] Page W.J., Cornish A. (1993). Growth of *Azotobacter vinelandii* UWD in fish peptone medium and simplified extraction of poly-β-hydroxybutyrate. Appl. Environ. Microbiol..

[21] Qu L., Ren L.J., Sun G.N., Ji X.J., Nie Z.K., Huang H. (2013). Batch, fed-batch and repeated fed-batch fermentation processes of the marine thraustochytrid *Schizochytrium* sp. for producing docosahexaenoic acid. Bioprocess Biosyst. Eng..

[22] Tanamool V., Enmak P., Kaewkannetra P. (2024). Batch fermentation of salt-acclimatizing microalga for omega-3 docosahexaenoic acid production using biodiesel-derived crude glycerol waste as a low-cost substrate. Fermentation.

[23] Ito T., Sota H., Honda H., Shimizu K., Kobayashi T. (1991). Efficient acetic acid production by repeated fed-batch fermentation using two fermenters. Appl. Microbiol. Biotechnol..

[24] Lu X., Li Y., Duan Z., Shi Z., Mao Z. (2003). A novel, repeated fed-batch, ethanol production system with extremely long term stability achieved by fully recycling fermented supernatants. Biotechnol. Lett..

[25] Baea S.M., Parka Y.C., Leea T.H., Kweona D.H., Choia J.H., Kimb S.K. (2004). Production of xylitol by recombinant *Saccharomyces cerevisiae* containing xylose reductase gene in repeated fed-batch and cell-recycle fermentations. Enzym. Microb. Technol..

[26] Lia C.Y., Chenb S.J., Chenga C.Y., Chena T.L. (2005). Production of *Acinetobacter radioresistens* lipase with repeated fed-batch culture. Biochem. Eng. J..

[27] Bauer R., Katsikis N., Varga S., Hekmat D. (2005). Study of the inhibitory effect of the product dihydroxyacetone on *Gluconobacter oxydans* in a semi-continuous two-stage repeated-fed-batch process. Bioprocess Biosyst. Eng..

[28] Chen S.Y., Wei Y.H., Chang J.S. (2007). Repeated pH-stat fed-batch fermentation for rhamnolipid production with indigenous *Pseudomonas aeruginosa* S2. Appl. Microbiol. Biotechnol..

[29] Khanna S., Srivastava A.K. (2005). Repeated batch cultivation of *Ralstonia eutropha* for poly (β- hydroxybutyrate) production. Biotechnol. Lett..

[30] Chala A.T., Matula S., Báťková K., Doležal F. (2019). Evaluation of methods for water and non-volatile LNAPL content measurement in porous media. Soil Water Res..

[31] Kumalaningsih S., Nur H., Nur A. (2011). Optimisation of polyhydroxyalkanoate (PHA) production from liquid bean curd waste by *Alcaligenes latus* bacteria. J. Agric. Food Technol..

[32] Grothe E., Moo-Young M., Chisti Y. (1999). Fermentation optimization for the production of (hydroxybutyric acid) microbial thermoplastic. Enzym. Microb. Technol..

[33] Yang X., Tsao G.T. (1995). Enhanced acetone-butanol fermentation using repeated fed-batch operation coupled with cell recycle by membrane and simultaneous removal of inhibitory products by adsorption. Biotechnol. Bioeng..

[34] Novak N., Gerdin S., Berovic B. (1997). Increased lovastatin formation by *Aspergillus terreus* using repeated fed-batch process. Biotechnol. Lett..

[35] Kumar M.S., Jana S.K., Senthil V., Shashanka V., Kumar S.V., Sadhukhan A.K. (2000). Repeated fed-batch process for improving lovastatin production. Process Biochem..

